# Functional movement analysis in patients with chronic nonspecific low back pain: a reliability and validity study

**DOI:** 10.1186/s12891-019-2779-6

**Published:** 2019-08-31

**Authors:** Johanna Vogel, Jan Wilke, Frieder Krause, Lutz Vogt, Daniel Niederer, Winfried Banzer

**Affiliations:** 1 Department of Sports Medicine, Goethe University Frankfurt/Main, Ginnheimer Landstraße 39, 60487 Frankfurt, Germany; 2 Institute for Occupational Medicine, Social Medicine and Environmental Medicine, University Hospital Frankfurt, Theodor-Stern-Kai 7, 60590 Frankfurt, Germany

**Keywords:** Low back pain, Unspecific pain, Idiopathic pain, Movement patterns, Kinematic analysis, Disability, Pain intensity

## Abstract

**Background:**

Individuals afflicted with nonspecific chronic low back pain (CLBP) exhibit altered fundamental movement patterns. However, there is a lack of validated analysis tools. The present study aimed to elucidate the measurement properties of a functional movement analysis (FMA) in patients with CLBP.

**Methods:**

In this validation (cross-sectional) study, patients with CLPB completed the FMA. The FMA consists of 11 standardised motor tasks mimicking activities of daily living. Four investigators (two experts and two novices) evaluated each item using an ordinal scale (0–5 points, one live and three video ratings). Interrater reliability was computed for the total score (maximum 55 points) using intra class correlation and for the individual items using Cohen’s weighted Kappa and free-marginal Kappa. Validity was estimated by calculating Spearman’s Rho correlations to compare the results of the movement analysis and the participants’ self-reported disability, and fear of movement.

**Results:**

Twenty-one participants (12 females, 9 males; 42.7 ± 14.3 years) were included. The reliability analysis for the sum score yielded ICC values between .92 and.94 (*p* < .05). The classification of individual scores are categorised ‘slight’ to ‘almost perfect’ agreement (.10–.91). No significant associations between disability or fear of movement with the overall score were found (*p* > .05). The study population showed comparably low pain levels, low scores of kinesiophobia and disability.

**Conclusion:**

The functional movement analysis displays excellent reliability for both, live and video rating. Due to the low levels of disability and pain in the present sample, further research is necessary to conclusively judge validity.

## Background

Chronic low back pain (CLBP) is a major health burden with a life time prevalence up to 84% [1]. The pathogenesis of CLBP is multifactorial. The symptoms can origin from several anatomical structures including nerve roots, intervertebral disks, muscles, fasciae, and bones [2, 3] as well as from psychological factors, such as stress, depression or anxiety [4]. More detailed, neuromuscular factors (i.e. deficits or impairments) are particularly named as risk factors and contributors to CLBP [5, 6]. Unlike the pain symptoms (as they are patient self-reported), neuromuscular contribution to CLBP may be assessed objectively.

While it is unclear whether they represent another potential (neuromuscular) cause or a consequence of the disorder, aberrations of fundamental movement patterns have been observed in patients with CLBP [7, 8]. Yet, all these reports [7, 8] focused on one particular joint or movement only. Systematic functional movement analyses, capturing fundamental movement patterns representing activities of daily living, might thus be a valuable addition to instrumental diagnostics like radiography and magnet resonance imaging [8]. In a previous trial, Wilke and Buhmann [3] showed that a functional movement analysis (FMA) could discriminate movement patterns of healthy individuals and patients with CLBP. The latter achieved considerably lower scores, reflecting worse movement quality, and increased side-to-side asymmetry when compared to a control group [3]. Despite these intriguing findings, the authors presented only a pilot evaluation of reliability. Therefore, our trial aimed to more thoroughly evaluate the measurement properties of the tool. This was done in two ways by assessing reliability in view of rater experience, assessment modes (live vs. video) and cross-validation to established subjective self-reported measures.

## Methods

### Ethical standard and study design

The study was approved by a local ethics committee and conducted in accordance to the ethical standards set by the declaration of Helsinki with its recent modification of Fortaleza [9, 10]. Each participant signed informed consent prior to study enrolment.

### Sample

Adults with CLBP were recruited. Recruitment strategies included posting of flyers (public) and personal addressing (outpatient rehabilitation centre) through the investigators. Participants were considered eligible if they fulfilled the following criteria: (1) chronic (> 13 weeks/3 months) nonspecific low back pain and (2) age from 18 to 65 years. Exclusion criteria comprised (1) severe psychiatric, neurological or cardiovascular diseases; (2) orthopaedic disorders except for low back pain; (3) pregnancy; (4) acute infectious disease and (5) intake of painkilling drugs, analgesics or muscle relaxants within the previous 48 h.

### Movement analyses

All participants performed the 30-min functional movement analysis [3] on two separate days. In between wash-out period was 1 week. The test consists of eleven movement tasks picturing activities of daily living (Table 1). For all the individual items, three repetitions were performed and each was rated. The best from these three was used for analysis. Unilateral, non-symmetrical tasks (e.g. lunge) were performed on both body sides. To ensure uniform testing conditions, all analyses were instructed by the same investigator. Standardised verbal commands as well as photo illustrations were used.

**Table 1:** Description of the eleven items of the movement analysis

Item	Starting position	Movement	Simplification
Thoracic mobility	Sit in a tall position on a chair with feet shoulder-width, place the short stick across the upper traps directly in front of the hip and hold the long stick with both hands on the right and left collarbone	Rotate your shoulders and trunk right/left while keeping your hip and feet still	
Shoulder mobility	Stand in a tall position with feet together and toes pointing forward, fists your hands while fingers surround the thumbs	Move your right/left fist over your head downwards and your other fist dorsal upwards, your hands don’t touch the back until you reach the end position	
Lifting Squat	Stand in a tall position with feet shoulder-wide and toes pointing forward, the long stick is hold with both hands, remove the stick on hip height until it’s on the height of the foot points	Squat down, leave your trunk straight vertical and heels on the ground during the whole movement	Using the board for heel support
Inline Lunge	Place on foot on the test board (toes on zero) and the other heel on your tibia lengths (measured before starting the screen), toes pointing forward and feet completely touch the board, the long stick is vertically contacted to your spine, while one hand hold the stick at the height of your cervical spine and the other on your lumbar spine, the long stick must touch your back of the head, your upper and lower back	Hold a straight trunk position, go down in the lunge position, your knee touches the board and the long stick holds the contact points during the whole movement	
Hurdle step	Stand in a tall position with feet together, toes pointing forward and touch the test kit, the long stick lies straight in your nape across your shoulders	Step with one leg over the hurdle, your heel touches the ground; come back in the starting position; during the whole movement the stick remains stable on your shoulders, your ankle joint, knee and hip build a straight line	
Forward bending	Stand in a tall position with feet shoulder-wide and toes pointing forward, you hold the long stick shoulder wide and touch your back of the head, bend your knees in a 135° angle and move your trunk in a horizontal position	Move the arms with the stick horizontal to the front until the arms are fully stretched, during the whole movement don’t move the rest of the body	
Pelvic stability	Go in the upper Push-up position, hands are vertically below your shoulders and your feet and shin bones build a right angle	elevate one leg until it is parallel to ground, then flex this knee of under the body as far as possible cranial, go back in the stretched position and following to the starting position	
Rotary stability	Go in the four-footed stand, your hands and knees are closed together, your toes are set up, your knees are vertically below your hip and your hands are vertically below your shoulders	Your arm and diagonal leg raised at the same time and build a “board”, then touch the elbow with the knee under your body, following go back first in the stretched position and then in the starting position; during the whole movement keep your back straight	Hands shoulder wide and knees hip wide
Dynamic Side plank	Go in the side plank position, your elbow is directly under your shoulder, your body forms a straight line, only the front part of your lower foot touches the ground, hold the long stick over the body vertically to ceiling	Lead stick with stretched arm on shoulder height before your body till the tip of stick touches the ground, go back in the starting position, during the whole movement don’t move the rest of your body	Knee angled
Push-Up	Go in a prone lying position, your hands are shoulder wide and your thumbs on height of your eyebrows (men)/ your chin (women), the feet are together, your toes are set up and your elbow and knees don’t touch the ground	Move your body upwards in one single flowing movement into the upper push-up position, during the whole movement hold your body stable	thumbs on height of chin (men) or shoulders (women)
Pull-Up	Go in a supine lying position under the pull-up bar, the bar is vertically over your chin (men)/ your shoulders (women), your feet are closed together	Pull your body upwards in one single flowing movement, during the whole movement hold your body stable	set feet up, knees are bent

**Fig. 1:**
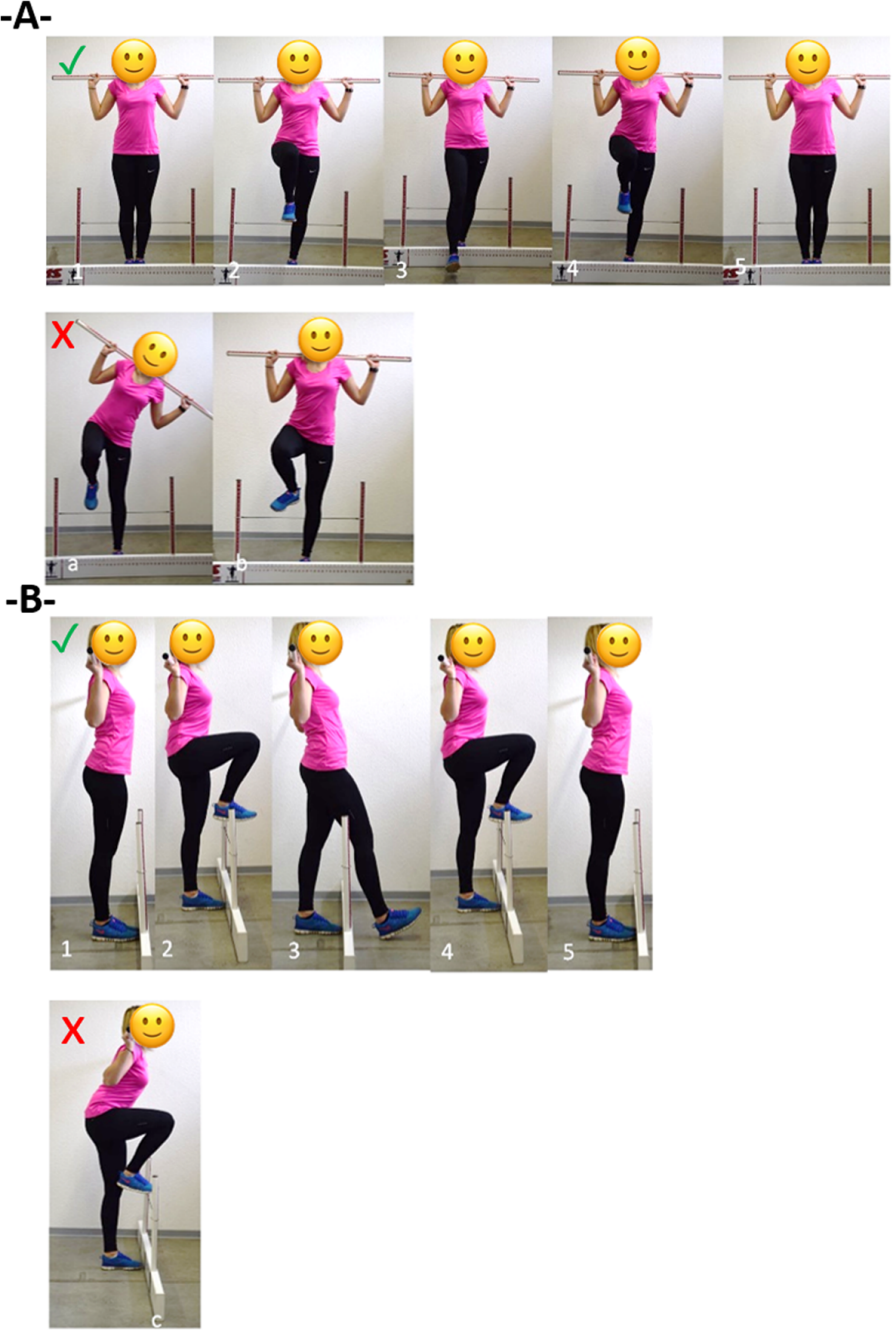
1.-5. correct performance of the test item Hurdle Step from frontal and sagittal plane; error patterns: **a**. hip/upper body get out to standing leg side, **b**. knee/ankle joint goes outwards, **c**. back is not straight at whole movement

For each of the 11 test items, performance was rated by means of an ordinal 6-point-Likert scale (0 to 5 points). Scoring was based on the identification of predefined error patterns indicating a lack of joint mobility or stability within the respective tasks (Fig. 1). If no compensatory movements were observed and the task was completed with high precision, the maximum value of five points per item was awarded. In contrast, each observed error pattern led to the deduction of one point. Thus, one error led to four, two errors to three and three errors to two points. A task was scored with one point, if more than three errors became manifest or the participant was unable to execute the requested movement. When reporting pain, zero points were documented, regardless of the error count. In eight of the 11 items, a predefined, simplified version was to be completed if a score of 5 was not achieved. In this case, a maximum of 4 points were obtainable. Again, but this time starting from the score of 4, each observed error pattern led to the deduction of one point.

#### Outcomes

##### Functional movement quality

Live and video ratings were conducted. The former was done by the investigator instructing the participants. For video rating, all analyses were captured from the frontal and sagittal plane, using two high-resolution cameras (HDR-CX240, Sony, Minato, Tokio), according to the procedures recommended in previous investigations [3]. Three raters independently evaluated the videos and one novice rater scores live. One of the video raters was a novice in scoring of non-apparative movement analyses, while the other two, classified as experts, had long-standing experience with the assessment and the observation of functional movement patterns. Prior to study initiation, the two novice raters received a detailed training on the use of the tool including demo scoring from the expert raters.

The total score was calculated by cumulating the individual scores of all 11 items. The highest achievable result was 55 points. In addition, the number of side-to-side asymmetries was documented. An asymmetry was defined as an unequal item score between left and right in the 7 non-symmetrical items. The maximum number of asymmetries, hence, was 7.

##### Self-reported function and disability

In addition to movement quality, psychometric data on self-reported function and disability were collected. To capture the levels of physical activity during the 7 day prior to study initiation, the participants completed the short form of the International Physical Activity Questionnaire (IPAQ) questionnaire (IPAQ-SF) [11]. With its seven questions, the IPAQ-SF assesses the number of days spent with intensive, moderate, and light activity, as well as the days characterized by sedentary behaviour. The outcome variable used for analysis was the overall level of activity in MET-minutes per week. The instrument has been demonstrated to display sufficient measurement properties [12, 13].

The pain intensity and the subjective disability of the patients was measured with the Chronic Pain Grade Questionnaire (CPGQ), developed by Korff et al. (1992) [14]. The six items of the questionnaire generate the data, each using an 11-point Likert-ordinal scale (ranging from 0 to 10). Three questions are based on pain intensity and three are based on subjective disability. Additionally, the number of days with disability during the past 3 months was asked. The pain intensity sum score and the disability sum score are built by means of z-transfomations of the original Likert scale questions on disability (3 questions) and pain (3 questions). Sum scores can range from 0 to 100 points. Based on these two scores, the participants were stratified according to the following severities of chronic pain: 0 = no pain in the last 3 months; I = low disability and low pain intensity; II = low disability and high pain intensity; III = high disability and moderate limiting; IV = high disability and severely limiting. An evaluation of the German version of the CPGQ shows that the questionnaire is a reliable and valid instrument for the rating of chronic pain severity [15].

To evaluate functional disability in performing activities of daily living, the Quebec Pain Disability Scale (QBPDS) was applied. Twenty questions addressing the capacity to perform activities of daily living were to be answered and scored on 6-point scales (0 = not difficult at all; 1 = marginally difficult; 2 = somewhat difficult; 3 = fairly difficult; 4 = very difficult; 5 = impossible to perform). The questionnaire is based on six sum categories: sleeping/rest (question 1–3); sitting/standing (question 4–6); locomotion (question 7–9); movement (question 10–12); bending forward (question 13–16) and carry heavy materials (question 17–20). The internal consistence of this questionnaire is good for the sum score (Cronbach’s alpha = .94), test-retest reliability (ICC = .81) has been shown to be high [16].

Pain-related fear of movement and injury was measured by means of the Tampa Scale of Kinesiophobia. A validated German version (TSK-GV) with eleven items to be scored on a 4-point Likert scale (strongly disagree to strongly agree) was used. The TSK-GV has been demonstrated to exhibit high reliability and validity [17].

### Statistics

#### Reliability

Reliability of the total score estimated by means of intraclass correlation coefficients (ICC 2.1). Reliability was analyzed twice, once for interrater (expert vs. novice; live vs. video rating) and once for intrarater (test-retest-design) agreement. According to Fleiss (1999) [18], resulting values were interpreted as ‘poor’ (ICC < .4), ‚fair to good’ (ICC .4–.75) and ‚excellent’ (ICC > .75).

To judge the concordance of the individual item ratings, Cohen’s weighted kappa statistics were used. Reliability of the side-to-side asymmetry rating was calculated with Free Marginal Kappa statistics [19]. The interpretation of all Kappa values was based on the recommendations of Landis and Koch (1977): k < 0 (‘poor agreement’); k = 0–0.20 (‘slight agreement’); k = 0.21–0.40 (‘fair agreement’); k = 0.41–0.60 (‘moderate agreement’); k = 0.61–0.80 (‘substantial agreement’); k = 0.81–1.00 (‘almost perfect agreement’). Every correlation was evaluated against the most experienced expert rater.

#### Validity

Systematic associations between movement pattern quality (assed via the FMA) and subjective disability (QBPDS), pain intensity (Korff’s CPG) as well as fear of movement (TSK scale) were examined using Spearman-Rho correlations. The significance level for all analyses was set to α = .05. All statistical calculations were done with SPSS 22.0 (SPSS Inc., Chicago, IL. USA).

## Results

No participant had to be excluded after study enrolment, no participant withdrawed his/her consent. Twenty-one participants (females = 12; males = 9 43 ± 14 years) were included. Psychometric data are displayed in Table 2. Overall, the sample displayed considerably low levels of pain, disability and kinesiophobia. The mean sum score achieved in the FMA was 31.0 ± 6.2 points. Per average, 2.5 ± 1.3 asymmetries were detected.

**Table 2:** Descriptive statistics of anthropometric data (age, BMI) and sum scores of the questionnaires

	Total
BMI (kg/m^2)^	22.7 (± 2.7)
7-Day Activity (MET-MIN)	3888 (± 3656)
Disability (QBPDS, 0–100 POINTS)	15 (± 11)
Fear of Movement (TSK, 11–44 POINTS)	19.15 (± 5.4)
Pain Intensity (KORFF, 0–100 POINTS)	34.1 (± 18)
Subjective Disability (KORFF,0–100 POINTS)	22.5 (± 22)

### Reliability

#### Interrater-reliability

The total score’s ICC values for the interrater reliability ranged between .92 and .94. The expert rater with video rating reached the highest ICC, the interrater correlation of the novice video rater was 0.93 and live rating reached the lowest ICC value.

The concordance of the individual scores are categorized in ‘slight agreement’ to ‘almost perfect agreement’. On average, three of the eleven items showed a fair (squat, rotary stability, side plank), three a moderate (inline lunge, forward bending, pelvic stability), three a substantial (hurdle step, push-up, pull-up) and two an almost perfect agreement (thoracic mobility, shoulder mobility) (Table 3).

**Table 3:** Weighted-kappa statistics of single items; k > 0.2 = (+), 0.2 > k > 0 = (o), k < 0 = (−)

Items	novice (live) vs. expert (video)		novice (video) vs. expert (video)		expert (video) vs. expert (video)	
	kappa	agreement	kappa	agreement	Kappa	agreement
Thoracic Mobility	.90 (+)	76%	.88 (+)	71%	.91 (+)	81%
Shoulder mobility	.87 (+)	81%	.83 (+)	76%	.86 (+)	81%
Squat	.34 (+)	38%	.40 (+)	38%	.41 (+)	52%
Inline Lunge	.51 (+)	33%	.34 (+)	19%	.63 (+)	47%
Hurdle step	.75 (+)	43%	.57 (+)	31%	.79 (+)	71%
Forward bending	.61 (+)	29%	.50 (+)	24%	.65(+)	57%
Pelvic stability	.69 (+)	62%	.49 (+)	38%	.10 (o)	29%
Rotary stability	.10 (o)	38%	.45 (+)	33%	.14 (o)	38%
Side plank	.37 (+)	19%	.36 (+)	33%	.39 (+)	28%
Push-Up	.66 (+)	38%	.74 (+)	52%	.91 (+)	81%
Pull-Up	.73 (+)	67%	.62 (+)	43%	.58 (+)	38%

The Free Marginal Kappa values for the ratings of movement asymmetry are displayed in Table 4. The agreement between the raters regarding asymmetries ranged from ‘poor’ to ‘substantial’ agreement. On average, the seven items reached once ‘poor’ (side plank), once ‘slight’ (inline lunge), three times ‘fair’ (hurdle step, pelvic stability, rotary stability), once ‘moderate’ (thoracic mobility) and once ‘substantial’ (shoulder mobility) correspondence.

**Table 4:** Free-Marginal kappa statistics of asymmetries; k > 0.2 = (+), 0.2 > k > 0 = (o), k < 0 = (−)

Items	novice - live vs. expert		novice - video vs. expert		expert - video vs. expert		Test-retest live	
	kappa	agreement	kappa	agreement	Kappa	agreement	Kappa	agreement
Thoracic Mobility	.33 (+)	66%	.62 (+)	80%	.62 (+)	80%	.20 (o)	60%
Shoulder mobility	.62 (+)	80%	.90 (+)	95%	.62 (+)	80%	.30 (+)	65%
Inline Lunge	.14 (o)	57%	−.05 (−)	47%	−.05 (−)	47%	.20 (o)	60%
Hurdle step	.52 (+)	76%	−.24 (+)	38%	.62 (+)	80%	.70 (+)	85%
Pelvic stability	.62 (+)	80%	.14 (o)	57%	.24 (+)	61%	.50 (+)	75%
Rotary stability	.24 (+)	61%	.62 (+)	80%	.14 (o)	57%	.20 (o)	60%
Side plank	−.24 (−)	38%	−.14 (−)	42%	−.05 (−)	47%	−.10 (−)	45%

#### Intrarater-reliability

The total score ICC value for the intrarater reliability is .91. The Free Marginal Kappa values show an agreement between the raters regarding ratings of the movement asymmetry ranged from ‘poor’ to ‘substantial’ agreement. The corresponding values are displayed in Table 4.

### Validity

No significant associations between total sum score of the functional movement analysis and measures of subjective movement disability or fear of movement were detected (*p* > .05; Table 5).

**Table 5:** Spearman-Rho statistics of the sum score of the functional movement analysis and subjective outcomes: Disability; Fear of movement; Pain intensity and subjective disability

	Correlation coefficient (r)	*p*-value
Disability (QBPDS, 0–100 POINTS)	−.206	.369
Fear of Movement (TSK, 11–44 POINTS)	−.259	.258
Pain Intensity (KORFF, 0–100 POINTS)	.006	.980
Subjective Disability (KORFF,0–100 POINTS)	−.089	.702

## Discussion

Our results show excellent values for interrater (video as well as live rating) and intrarater reliability (live rating) for the functional movement analysis. The interrater correspondence of the eleven individual items reached from ‘slight’ to ‘almost perfect’ agreement. Six of seven items with asymmetries reached ‘slight’ to ‘substantial’ agreement for both inter- and intrarater reliability. More experience in the use of FMA resulted in an only minimally improved accordance. No correlations between the subjective outcomes and functional movement analysis were identified.

Previous studies investigating the reliability of other systematic movement analysis approaches (e.g. the Functional Movement Screen, FMS) show inconsistent results (ICCs ranging from 0.38 to 0.92) [20–22]. A systematic review found a mean ICC of 0.81 for interrater reliability of the FMS [23]. The overall reliability in the present FMA is higher compared to these other findings. Also, the individual items in the present FMA showed a better reliability than the FMS: substantial reliability (k ≥ 0.4) in 72% (8/11) compared to only 57% (4/7) [24].

In other visual movement analyses, good interrater reliability values were reached when different degrees of experience (expert versus novice) and assessment modes (live versus video rating) were compared. In a movement analysis designed to investigate the lower limbs with a standardised procedure, good interrater reliability (trained beginners vs. experts) values were reached [25]. Similar results were found for the FMS: Trained novice raters were objective compared to expert raters [20]. These findings are in line with the results of the present FMA and support our findings.

Other studies comparing live versus video rating showed varying reliability, ranging from poor (ICC = .23) to excellent (ICC = .92) [21, 26]. Our results support the latter. In the present study, the videos were recorded from the frontal and the sagittal plane to cover the entire movement. Advantages of this video-based evaluation includes the possibility to watch movements as often as necessary and at different velocities as well as pausing the video at critical positions. The excellent reliability in the present FMA between live and video rating indicates that the described standardised video recording with two cameras from two different perspectives is successful.

We found no significant associations between total score of the functional movement analysis and measures of subjective movement disability or fear of movement. One previous study demonstrated that the movement analysis can discriminate between back pain patients and healthy individuals [3]. Patients show a lower sum-score and display more movement asymmetries. The patients with back pain in the study of Wilke and Buhmann (2013) [3] reached a mean sum score of 32.0 points and had 3.8 asymmetries. The population of the present study reached comparable values in sum-score of 31 points, but showed considerably fewer asymmetries of 2.5 points. Reasons for the finding of no association may be found in (1) the low subjective movement disability and pain intensity levels in our participants, in particular when compared to other study populations with CLBP [16, 17], (2) in the low variability of the data (QBPDS values ranging only from 0 to 36 of possible 0–100 points), and (3) a high physical activity of the participants. The study population showed a mean amount of physical activity of 3888 MET-minutes in the week before study inclusion. This can be interpreted as a very high activity level [9]. One may speculate that active people with chronic back pain do not change their movement behavior as strongly as their inactive counterparts. Therefore, they experienced only a low subjective movement disability in their everyday life. In any case, as physical activity, to a certain extent, is associated with reduced back pain and movement disability [27], this might also explain the lack of correlations. Further research should, therefore, aim to examine if associations of movement quality and self-reported parameters of pain and function become manifest in patients with increased disability and movement fear.

The present findings have implications for clinical practice. So far, systematic visual investigations of functional movement patterns have only been implemented in competitive sports [28, 29]. As alterations of fundamental movement patterns have been demonstrated in patients with low back pain [8, 30], the present tool may be an interesting addition to the available pool of diagnostic instruments. The present FMA shows a good interrater reliability and can be confidently used by both novice and experienced raters using live and video scoring. It provides a straightforward estimate of fundamental movement quality. Regarding to the validity, the FMA could discriminate between patient and healthy adults [3], but could not distinguished between different disability levels in participants with a low overall level of pain. Consequently, and as our target population did not indicate pain during movements, it can safety be applied in participants with chronic pain, yet its discrimination validity is at least questionable. Nonetheless, further research, not displaying the limitation of patients with low disability levels, limited pain and high physical activity, should be conducted in order to conclusively judge its value regarding an association with self-reported measures. Moreover, future studies are warranted to examine whether determined task failures due to impaired movement patterns can be addressed and improved by particular correction exercises.

## Conclusion

The standardised functional movement analysis displays a good interrater-reliability when different education degrees and observation methods are compared. The functional movement is usable in patients with chronic low back pain but it remains unclear whether the movement analysis can identify different degrees of subjective disability in tasks of daily living and should be clarified in additional studies.

## Data Availability

The datasets used and analysed during the current study are available from the corresponding author on reasonable request.

## Abbreviations

CLBP: Chronic Low Back Pain
CPG: Chronic Pain Grade
FMA: Functional Movement Analysis
FMS: Functional Movement Screen
ICC: Intraclass Correlation Coefficient
IPAQ-SF: International Physical Activity Questionnaire- Short Form
MET: Metabolic Equivalent of Task
QBPDS: Quebec Back Pain Disability Scale
TSK-GV: Tampa Scale of Kinesiophobia – German Version
